# Extravascular lung water levels are associated with mortality: a systematic review and meta-analysis

**DOI:** 10.1186/s13054-022-04061-6

**Published:** 2022-07-06

**Authors:** Francesco Gavelli, Rui Shi, Jean-Louis Teboul, Danila Azzolina, Pablo Mercado, Mathieu Jozwiak, Michelle S. Chew, Wolfgang Huber, Mikhail Y. Kirov, Vsevolod V. Kuzkov, Tobias Lahmer, Manu L. N. G. Malbrain, Jihad Mallat, Samir G. Sakka, Takashi Tagami, Tài Pham, Xavier Monnet

**Affiliations:** 1 Service de Médecine Intensive-Réanimation, AP-HP, Hôpital de Bicêtre, DMU CORREVE, 78, Rue du Général Leclerc, 94 270 Le Kremlin-Bicêtre, France; 2 Emergency Medicine Unit, Department of Translational Medicine, Università degli Studi del Piemonte Orientale, Novara, Italy; 3 Université Paris-Saclay, Inserm UMR S_999, FHU SEPSIS, CARMAS, Le Kremlin-Bicêtre, France; 4 Department of Environmental and Preventive Science, University of Ferrara, Ferrara, Italy; 5 Facultad de Medicina Clínica Alemana, Universidad del Desarrollo, Santiago, Chile; 6 Service de Médecine Intensive Réanimation, Centre Hospitalier Universitaire l’Archet 1, 151 route Saint Antoine de Ginestière, 06200 Nice, France; 7 Equipe 2 CARRES, UR2CA - Unité de Recherche Clinique Côte d’Azur, Université Côte d’Azur, Nice, France; 8 Department of Anaesthesia and Intensive Care, Biomedical and Clinical Sciences, Linköping University, Linköping, Sweden; 9 II. Medizinische Klinik Und Poliklinik, Klinikum Rechts Der Isar der Technischen Universität München, Munich, Germany; 10 Department of Anesthesiology and Intensive Care Medicine, Northern State Medical University, Arkhangelsk, Russia; 11 First Department Anaesthesiology and Intensive Therapy, Medical University of Lublin, Jaczewskiego Street 8, 20-954 Lublin, Poland; 12 International Fluid Academy, Lovenjoel, Belgium; 13 Department of Anesthesiology and Critical Care Medicine, Schaffner Hospital, Lens, France; 14 Department of Critical Care Medicine, Cleveland Clinic Abu Dhabi, Abu Dhabi, United Arab Emirates; 15 Department of Intensive Care Medicine, Gemeinschaftsklinikum Mittelrhein gGmbH, Academic Teaching Hospital of the Johannes Gutenberg University Mainz, Koblenz, Germany; 16 Department of Emergency and Critical Care Medicine, Nippon Medical School Musashi Kosugi Hospital, Kawasaki, Kanagawa Japan; 17 Université Paris-Saclay, UVSQ Inserm U1018, Equipe d’Epidémiologie Respiratoire Intégrative, CESP, 94807 Villejuif, France

**Keywords:** Lung edema, Transpulmonary thermodilution, Hemodynamic monitoring, Critically ill patients

## Abstract

**Background:**

The prognostic value of extravascular lung water (EVLW) measured by transpulmonary thermodilution (TPTD) in critically ill patients is debated. We performed a systematic review and meta-analysis of studies assessing the effects of TPTD-estimated EVLW on mortality in critically ill patients.

**Methods:**

Cohort studies published in English from Embase, MEDLINE, and the Cochrane Database of Systematic Reviews from 1960 to 1 June 2021 were systematically searched. From eligible studies, the values of the odds ratio (OR) of EVLW as a risk factor for mortality, and the value of EVLW in survivors and non-survivors were extracted. Pooled OR were calculated from available studies. Mean differences and standard deviation of the EVLW between survivors and non-survivors were calculated. A random effects model was computed on the weighted mean differences across the two groups to estimate the pooled size effect. Subgroup analyses were performed to explore the possible sources of heterogeneity.

**Results:**

Of the 18 studies included (1296 patients), OR could be extracted from 11 studies including 905 patients (464 survivors vs. 441 non-survivors), and 17 studies reported EVLW values of survivors and non-survivors, including 1246 patients (680 survivors vs. 566 non-survivors). The pooled OR of EVLW for mortality from eleven studies was 1.69 (95% confidence interval (CI) [1.22; 2.34], *p* < 0.0015). EVLW was significantly lower in survivors than non-survivors, with a mean difference of −4.97 mL/kg (95% CI [−6.54; −3.41], *p* < 0.001). The results regarding OR and mean differences were consistent in subgroup analyses.

**Conclusions:**

The value of EVLW measured by TPTD is associated with mortality in critically ill patients and is significantly higher in non-survivors than in survivors. This finding may also be interpreted as an indirect confirmation of the reliability of TPTD for estimating EVLW at the bedside. Nevertheless, our results should be considered cautiously due to the high risk of bias of many studies included in the meta-analysis and the low rating of certainty of evidence.

*Trial registration* the study protocol was prospectively registered on PROSPERO: CRD42019126985.

**Supplementary Information:**

The online version contains supplementary material available at 10.1186/s13054-022-04061-6.

## Background

Extravascular lung water (EVLW) represents the amount of lung fluid outside the pulmonary vasculature, i.e. the cellular and extracellular fluid volume of the interstitial and alveolar spaces [1, 2]. As such, its elevation is an important pathophysiological pattern of hydrostatic pulmonary edema and acute respiratory distress syndrome (ARDS) [3]. The level of EVLW is correlated with the degree of diffuse alveolar damage in patients with ARDS [4].

Today, transpulmonary thermodilution (TPTD) is the only technique that allows the estimation of the total amount of EVLW [2]. This estimation has been validated against gravimetry, which is the reference method, in an autopsy study in humans [5]. It has been shown that TPTD is able to detect small and rapid increases in EVLW [6], contributing to the validation of the method.

Several studies have investigated the relationship between the amount of EVLW and mortality in septic patients [7], patients with ARDS [8] and critically ill patients in general [9]. Nevertheless, many of these studies were of small size [10, 11], some were retrospective [9, 12] and the link between EVLW and mortality reported by some of them was weak [13, 14]. A previous meta-analysis on the association of EVLW and mortality was performed ten years ago [15]. Nevertheless, it included studies in which EVLW had been evaluated through the double-indicator technique, which is not used anymore. Moreover, several other studies have since been performed. The relationship between the value of EVLW and outcome remains an important question. Confirming the prognostic value of EVLW may reinforce the clinical interest of the variable [16]. In addition, if it exists, it may indirectly contribute to confirming the reliability of its estimation by TPTD.

## Methods

### Clinical research question

The clinical research question was: What is the relationship between EVLW and mortality in critically ill patients?

### PICO statement

The PICO statement was the following:

- P-patient, problem or population: Critically ill adult patients.
- I-intervention or exposure: Measurement of EVLW through the single indicator transpulmonary dilution method.
- C-comparison, control or comparator: Comparison of EVLW between survivors and non-survivors patients, considering either the baseline value or maximal value reached during the intensive care unit (ICU) stay.
- O-outcome: The primary outcome was the odds ratio (OR) of EVLW as a risk factor for mortality, defined either as in-hospital or 28-day or ICU mortality. The secondary outcome was mean differences between survivors and non-survivors in terms of EVLW value.

### Identification of records

Our aim was to identify all studies evaluating the association between EVLW measured by TPTD, whatever the threshold used to define an elevated EVLW, and mortality in critically ill patients. We included in our analysis only studies that were published in full text or accepted for publication in indexed journals.

We searched the US National Library of Medicine’s MEDLINE database, the Embase database, and the Cochrane Database of Systematic Reviews for relevant studies published from 1960 to 1 June, 2021. We used the following medical subject headings and keywords: ‘‘EVLW”, “EVLWi”, “lung water”, “survival”, and “mortality”. The complete searching strategy is reported in Additional file 1: S1. We also looked for relevant articles cited in review articles, commentaries, editorials, and in the references of the original articles identified by our search. We excluded studies performed in children and in burned patients, studies published in languages other than English, and studies in which EVLW was estimated by methods different from TPTD. The search was performed by two authors (FG and RS) until no new records could be found. Conflicts regarding the inclusion or exclusion of studies were resolved by consensus with a third investigator (XM). The meta-analysis was performed according to the PRISMA statement [17] (Additional file 1: S2). The study protocol was prospectively registered in PROSPERO (CRD42019126985).

### Data extraction

Using a standardized data form, we extracted several data elements from the included studies, including characteristics of the investigated population, the method used to measure EVLW, and the timing at which EVLW was measured. We collected the OR with its 95% confidence interval (95% CI) of EVLW as a risk factor for mortality, if available. If data needed for the analysis were not retrievable from the text, tables or figures, we systematically asked them to the authors of the studies.

### Assessment of risk of bias in included studies

Two authors (FG and RS) independently assessed the overall quality of evidence at the outcome level according to the Grading of Recommendations, Assessment, Development, and Evaluation (GRADE) system [18]. Moreover, they assessed the risk of bias of the included studies by following the criteria specified in the QUIPS tool [19]. It should be noted that this tool was not the one we initially planned to use for assessing the risk of bias (PROSPERO: CRD42019126985). For each criterion, the risk of bias was judged as high, moderate, or low. Disagreements between the reviewers were resolved by consensus with a third investigator (XM).

### Statistical analysis

Pooled ORs were performed using continuity corrections [20]. Mean differences and standard deviation (SD) of the EVLW between survivors and non-survivors were considered. If a confidence interval of EVLW was reported, we converted it to SD for pooled analysis. The 95% CI was calculated using the Wilson method [21]. A random effects meta-analysis model was computed on the weighted mean differences (WMD) across the two groups to estimate the pooled size effect. A value of *I*^2^ ≥ 75% was considered as indicating a high heterogeneity [22].

To investigate the source of heterogeneity, pre-defined subgroup analyses were performed:

- Timing of EVLW measurement: baseline (≤ 48 h) versus maximal value
- EVLW indexation: actual versus predicted body weight
- Study population: ARDS versus non-ARDS
- Risk of bias: “moderate and low” versus “high”.

Publication bias was investigated using Deek’s test [23, 24]. The statistical significance was set at a *p* value < 0.05. The analyses were performed by using Review Manager version 5.3, R 3.3.5 with metafor packages [25].

## Results

### Characteristics of the included studies

**Fig. 1:**
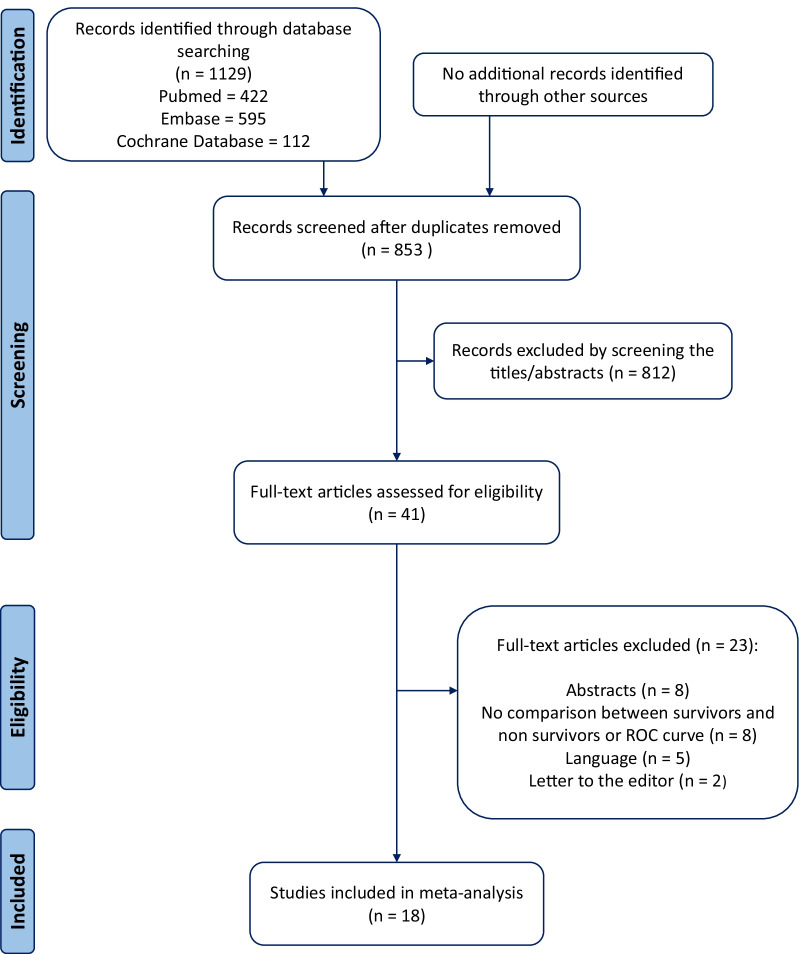
PRISMA flowchart

We included 18 studies that reported EVLW and mortality, with a total of 1296 patients enrolled [7, 8, 11–14, 26–37]. The flow chart is presented in Fig. 1. Data from nine studies [7, 8, 13, 14, 26, 31, 32, 36, 37] that were missing in the published articles were obtained by direct contact with authors, or retrieved in our database for studies performed by our group.

The main characteristics of the studies are reported in Table 1. Nine studies were performed specifically in ARDS patients [8, 11, 14, 30, 33–37], seven in septic shock patients [7, 12, 27–29, 31, 32], and two in unselected critically ill patients [13, 26]. All studies were performed in patients admitted to the ICU. Of them, the OR of EVLW as a risk factor for mortality could be extracted from 11 studies [8, 12, 13, 26, 28–30, 32, 33, 36, 37]. In 17 studies [7, 8, 11–14, 26–35, 37], the value of EVLW was provided at baseline, i.e. at the first time, it was measured (Table 1). The maximal value of EVLW observed during the study period was available in ten studies [7, 8, 12–14, 31, 32, 34, 36, 37], one in unselected critically ill patients [13], five in patients with ARDS [8, 14, 34, 36, 37] and four in patients with septic shock [7, 12, 31, 32] (Table 1).

**Table 1:** Main characteristics of included studies

Study ID	Year	No. of patients	Country	Type of study	Setting	Type of patient	EVLW indexation	Outcome
Martin et al. [27]	2005	29	USA	Prospective	Medical ICU	Severe sepsis/septic shock	ABW	28-Day mortality
Kuzkov et al. [7]	2006	38	Russia	Prospective	Mixed ICU	Septic shock/ALI	ABW	28-Day mortality
Chung et al. [28]	2008	33	Taiwan	Prospective	Medical ICU	Severe sepsis/septic shock	ABW	In-hospital mortality
Phillips et al. [11]	2008	19	USA	Prospective	ICU	ARDS	PBW/ABW	ICU mortality
Chung et al. [29]	2010	67	Taiwan	Prospective	Medical ICU	Severe sepsis/septic shock	PBW	ICU mortality
Craig et al. [30]	2010	44	UK	Prospective	ICU	ALI/ARDS	PBW/ABW	ICU mortality
Chew et al. [31]	2012	51	Sweden	Prospective	Mixed ICU	Severe sepsis/septic shock	PBW/ABW	ICU mortality
Cordemans et al. [13]	2012	123	Belgium	Retrospective	ICU	Critically ill	ABW	28-Day mortality
Mallat et al. [32]	2012	55	France	Prospective	Mixed ICU	Septic shock	PBW/ABW	ICU mortality
Brown et al. [33]	2013	59	UK	Prospective	ICU	ALI/ARDS	PBW	ICU mortality
Jozwiak et al. [8]	2013	200	France	Retrospective	Medical ICU	ARDS	PBW	28-Day mortality
Huber et al. [26]	2014	50	Germany	Prospective	ICU	Critically ill	PBW	ICU mortality
Tagami et al. [14]	2014	192	Japan	Post-hoc analysis	ICU	ARDS	PBW	28-Day mortality
Zhao et al. [34]	2015	21	China	Prospective	ICU	ARDS	PBW	ICU mortality
Wang et al. [12]	2016	105	China	Retrospective	ICU	Septic shock	PBW	28-Day mortality
Ma et al. [35]	2019	41	China	Retrospective	ICU	ARDS	PBW	In-hospital mortality
Huber et al. [36]	2020	49	Germany	Prospective	ICU	ARDS	PBW	28-Day mortality
Shi et al. [37]	2021	120	France	Prospective	ICU	ARDS	PBW	ICU mortality

*ABW* actual body weight, *ALI* acute lung injury, *ARDS* acute respiratory distress syndrome, *EVLW* extravascular lung water, *ICU* intensive care unit, *PBW* predicted body weight, *UK* United Kingdom, *USA* United States of America

Mortality was defined as the 28-day mortality in seven studies [7, 8, 12–14, 27, 36], as the ICU mortality in nine [11, 26, 29–34, 37], and as the in-hospital mortality in two studies [28, 35] (Table 1). The results of the GRADE and the QUIPS evaluation are provided in Table 2 and Additional file 1: S3.

**Table 2:** The results of the Grading of Recommendations, Assessment, Development, and Evaluation (GRADE) assessment of the evidence certainty on the association between the extravascular lung water and mortality

Outcome	Relative effect (95% CI)	No. of patients		No. of participants (studies)	Downgrade factors					Certainty of the evidence (GRADE) 18
		Survivors	Non-survivors		Risk of bias	Inconsistency	Imprecision	Indirectness	Publication bias	
Mortality	OR 1.69 (1.22–2.34)	464/905 (51.3%)	441/905 (48.7%)	905 Adult patients (11 studies)	Serious^a^	Serious^b^	Not serious	Serious^c^	Not serious	⊕○○○ VERY LOW
	WMD − 4.97 mL/kg (− 6.54; − 3.41)	680/1246 (54.6%)	566/1246 (45.4%)	1246 Adult patients (17 studies)	Serious^a^	Serious^b^	Not serious	Serious^c^	Not serious	⊕○○○ VERY LOW

*CI* confidence interval, *OR* odds ratio, *WMD* weighted mean difference

^a^Downgraded by one level for the risk of bias because ten of 18 studies were evaluated as high risk of bias according to the QUIPS tool and four of 11 studies did not report adjusted OR. Nevertheless, no differences regarding the relative effects were observed between high and moderate and low risk of bias in subgroup analysis

^b^Downgraded by one level for inconsistency: substantial heterogeneity is seen between studies (*I*^2^ > 75%)

^c^Downgraded by one level for indirectness because different cut-off values for elevated EVLW definition (> 7 mL/kg/m^2^ in two studies, > 10 mL/kg/m^2^ in eight studies, not available in eight studies)

### Association of EVLW with mortality

**Fig. 2:**
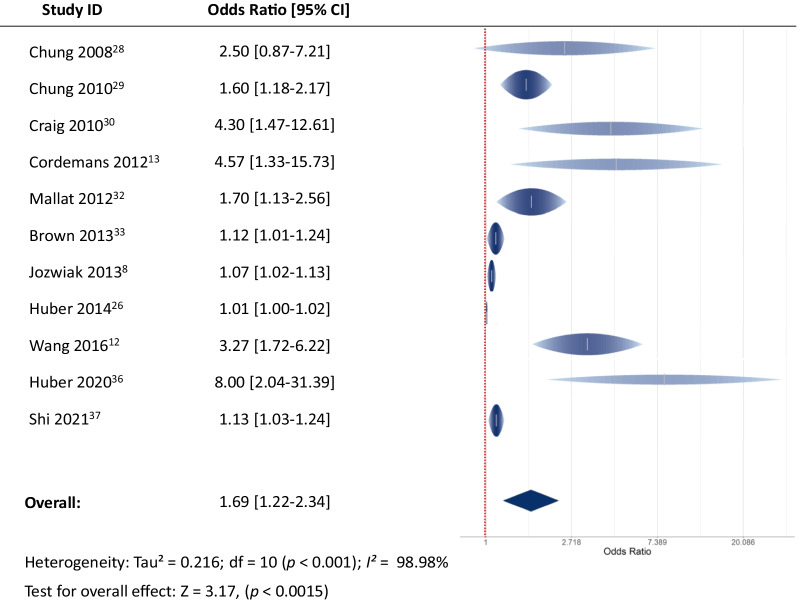
The pooled odds ratio of EVLW for mortality

The pooled OR obtained from the 11 studies that reported OR [8, 12, 13, 26, 28–30, 32, 33, 36, 37] was 1.69 (95% CI [1.22; 2.34], *I*^2^ = 98.98%, *p* < 0.0015) (Fig. 2). Seventeen studies reported EVLW values of survivors and non-survivors, including 1 246 patients (680 survivors vs. 566 non-survivors) [7, 8, 11–14, 27–37] (Additional file 1: S4). The weighted mortality rates are presented in Additional file 1: Figure S5.

**Fig. 3:**
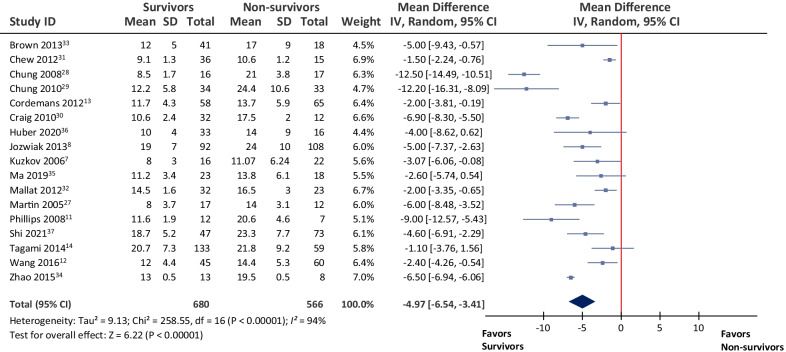
Mean difference in extravascular lung water levels between survivors and non-survivors

Overall, EVLW was significantly lower in survivors compared to non-survivors, with a mean difference of − 4.97 mL/kg (95% CI [− 6.54; − 3.41], *p* < 0.001) (Fig. 3). Since the statistical heterogeneity was significant (*I*^2^ = 93.8%, *p* < 0.001), the random-effects model was used to pool the data. The results in the prespecified subgroups were as follows.

#### Baseline EVLW versus maximal EVLW

When comparing OR of EVLW as a risk factor for mortality between studies in which EVLW at baseline was reported [28–30, 33, 36] to those in which maximal EVLW was reported [8, 12, 13, 26, 32, 37], the EVLW remained to be a risk factor in both groups (OR of group baseline EVLW: 2.22, 95% CI [1.17; 4.20] vs. OR of group maximal EVLW: 1.48, 95% CI [1.01; 2.17], *p* = 0.38) (Additional file 1: Figure S6).

In the eight studies in which the EVLW at baseline was reported [11, 27–31, 33, 35], it was lower in survivors than in non-survivors (WMD: − 6.90 mL/kg, 95% CI [− 10.27; − 3.53], *p* < 0.001). In the nine studies in which the maximal value of EVLW was reported [7, 8, 12–14, 32, 34, 36, 37], it was also lower in survivors than in non-survivors (WMD: − 3.43 mL/kg, 95% CI [− 5.28; − 1.59], *p* < 0.001). The WMD was not different between the two categories of studies (*p* = 0.08) (Additional file 1: Figure S7).

#### Actual versus predicted body weight for EVLW indexation

When comparing OR of EVLW as a risk factor for mortality between studies in which EVLW was indexed to actual body weight [13, 28, 32] to those in which it was indexed to predicted body weight [8, 12, 26, 29, 30, 32, 33, 36, 37], EVLW remained a risk factor in both groups (OR of actual body weight for EVLW indexation: 2.37, 95% CI [1.47; 3.83] vs. OR of predicted body weight for EVLW indexation: 1.54, 95% CI [1.13; 2.10], *p* = 0.16) (Additional file 1: Figure S8).

In the four studies that reported the EVLW indexed to actual body weight [7, 13, 27, 28], the survivors had significantly lower values of EVLW than non-survivors (WMD: − 5.92 mL/kg, 95% CI [− 11.09; − 0.75], *p* = 0.02). This was also the case in the 13 studies in which the EVLW was indexed to predicted body weight [8, 11, 12, 14, 29–37] (WMD: − 4.64 mL/kg, 95% CI [− 6.35; − 2.94], *p* < 0.001). The WMD was not different between the two groups (*p* = 0.65) (Additional file 1: Figure S9).

#### ARDS population versus non-ARDS population

When comparing OR of EVLW acting as a risk factor for mortality between studies that included ARDS patients [8, 30, 33, 36, 37] to those that included non-ARDS patients [12, 13, 26, 28, 29, 32], EVLW remained a risk factor in both groups (OR in ARDS patients: 1.09, 95% CI [1.05, 1.14] vs. OR in non-ARDS patients: 1.83, 95% CI [1.20, 2.79], *p* = 0.57) (Additional file 1: Figure S10).

In the nine studies dedicated to ARDS patients [8, 11, 14, 30, 33–37], the EVLW was lower in survivors than non-survivors (WMD: − 5.16 mL/kg, 95% CI [− 6.48; − 3.84], *p* < 0.001). This was also the case in the eight studies that included non-ARDS patients [7, 12, 13, 27–29, 31, 32] (WMD: − 5.00 mL/kg, 95% CI [− 7.65; − 2.35], *p* < 0.001). No significant difference in WMD was observed between the two groups (*p* = 0.92) (Additional file 1: Figure S11).

#### Risk of bias

When comparing studies according to the global risk of bias, there was no significant difference in OR between the studies with a high [26, 28, 30, 36] and moderate and low [8, 12, 13, 29, 32, 33, 37] risk of bias (OR of studies with low risk of bias: 1.46, 95% CI [1.10; 1.94] vs. OR in studies with a high risk of bias 2.62, 95% CI [1.04; 6.60], *p* = 0.37) (Additional file 1: Figure S12).

In studies with a moderate and low risk of bias [8, 12–14, 29, 32, 33, 37], the EVLW was lower in survivors than in non-survivors (WMD: − 3.80 mL/kg, 95% CI [− 5.49; − 2.11], *p* < 0.001). This was also the case in the studies with a high risk of bias [7, 11, 27, 28, 30, 31, 34–36] (WMD: − 5.83 mL/kg, 95% CI [− 8.12; − 3.54], *p* < 0.001) No significant difference in WMD was observed between the two groups, *p* = 0.16) (Additional file 1: Figure S13).

### Publication bias

According to the results of Deek’s test, the funnel plot asymmetry test revealed the absence of publication bias within the studies considered (*p* = 0.31) (Additional file 1: Figure S14).

## Discussion

Our systematic review and meta-analysis of 18 studies, involving 1296 patients, suggests that an increased value of EVLW is associated with increased mortality compared to less elevated values in ICU patients. The levels of EVLW were less increased in survivors compared to non-survivors. However, due to the high risk of bias of included studies and the low rating of certainty of evidence according to the GRADE assessment, these conclusions should be considered with caution.

A major advantage of TPTD, which is part of the advanced monitoring techniques in critically ill patients [38–41], is to provide a bedside estimation of EVLW [42]. EVLW measured by TPTD has been demonstrated to reliably detect diffuse alveolar damage (DAD), which is the histologic pattern of ARDS [4, 43–45]. The severity of DAD is heterogeneous among ARDS patients, and this is in accordance with the heterogeneity of EVLW in this population, as we have recently observed for instance in ARDS patients with Coronavirus disease 2019 (COVID-19) [37]. Since the presence of DAD is associated with a poorer outcome in ARDS [44, 45], EVLW may reflect the severity of pulmonary lesions in critically ill patients.

However, most of the conclusions regarding the prognostic value of EVLW come from heterogeneous studies, performed in different settings and with different methodologies. While some authors reported a close relationship between EVLW values and outcome [11], others did not [14]. Moreover, some studies included only a small series of patients [11, 34]. The present meta-analysis may thus clarify the relationship between EVLW and outcome in ICU patients. We found that an increased value of EVLW is one of the prognostic factors for mortality in ICU patients. The OR of EVLW as a risk factor for mortality was 1.69 [1.22; 2.34]. Also, mortality was significantly higher in patients with the highest EVLW values, either at baseline or at its maximum, compared to patients with the lowest EVLW values.

The heterogeneity of the included studies was significant. However, the subgroup analyses for OR and WMD were conducted to investigate the sources of heterogeneity. The association between EVLW and mortality has been described at different times, i.e. baseline, Day-3, or when it reached its maximal value, likely because these timings highly depend on the time when the TPTD device was set up. Nevertheless, our subgroup analyses showed that an increase in EVLW remains an unfavourable prognostic factor, regardless of the timing at which it is measured. In addition, we found no difference between studies in which EVLW was indexed to predicted body weight and those in which it was indexed to actual body weight, regarding OR for mortality as well as mean differences between survivors and non-survivors. Nonetheless, as the between-group difference between survivors and non-survivors was quite small and as the dimension of the lungs depends on the height of the patient rather than on actual weight fluctuations [26, 30], we still suggest indexing EVLW to the predicted body weight. EVLW was similarly associated with mortality in studies that specifically included ARDS patients and in studies with non-ARDS patients. This may suggest the value of EVLW for indicating disease severity not only in ARDS but also in other critically ill patients.

Since the risk of bias was estimated as high for many studies included and our results have “very low certainty of evidence” according to the GRADE assessment, our conclusions should be considered with caution. Obviously, EVLW should not be used to predict the outcome of ICU patients on an individual basis. There are many other prognostic factors in ICU patients. We rather believe that our results indirectly contribute to the recognition of TPTD for estimating EVLW. Indeed, EVLW measured by the technique would not be associated with the outcome if this estimation was unreliable. Although the estimation of EVLW by TPTD has been demonstrated to be correlated with the reference technique [46], reproducible [47], and able to detect small [48, 49] and rapid [6] variations, doubts may persist regarding its reliability [2, 3]. As the gold standard technique for measuring EVLW, namely gravimetry, can be performed only in cadavers, the validation of EVLW measurements in patients can only be indirect. The present meta-analysis may contribute to this indirect validation. Thus, our results suggest that clinicians may rely on the estimation of EVLW by TPTD. Besides, EVLW may help to identify patients with DAD and to grade the severity of ARDS [50, 51]. It may also be used in fluid management, as a marker indicating the risk of fluid administration, or as a guide for fluid removal [52]. Further studies should investigate the clinical interest of such strategies, describe the relationship between EVLW and respiratory mechanics, or evaluate the effect of some respiratory management such as prone position [53]. Studies testing the interest of integrating EVLW in the strategy of fluid management are also needed to better identify its clinical significance.

Our study suffers from many limitations. First, the OR of EVLW as a risk factor for mortality, which is the main factor to consider in meta-analyses on prognostic factors, was not provided in all the studies we included. Second, data for the comparison of the mean difference between survivors and non-survivors was not available in one of the included studies [26]. Nevertheless, this represents a minority (4%) of the whole cohort. Third, we did not obtain data regarding fluid balance since our principal objective was to confirm that EVLW measured by TPTD is associated with a worse outcome. Fourth, we did not investigate EVLW as an adjunctive variable to other techniques, such as ultrasonography and bioelectrical impedance, to evaluate the fluid status [54]. Finally, we limited our search to articles published in English language and did not expand our search to clinical trial registry databases.

## Conclusion

In conclusion, although limited by the low rating of certainty of the evidence, this meta-analysis suggests that elevated levels of EVLW measured by TPTD are associated with mortality in ICU patients. This finding may be interpreted as an indirect confirmation of the reliability of TPTD for estimating EVLW.

## Supplementary Information

**Additional file 1.** Supplementary information on further results.

## Data Availability

The datasets used and/or analysed in the present study are available from the corresponding author on reasonable request.

## Abbreviations

ARDS: Acute respiratory distress syndrome
EVLW: Extravascular lung water
GRADE: Grading of Recommendations Assessment, Development and Evaluation
ICU: Intensive care unit
OR: Odds ratio
TPTD: Transpulmonary thermodilution
WMD: Weighted mean difference
