# Supplementary material for: Extravascular lung water levels are associated with mortality: a systematic review and meta-analysis
Source: Crit Care. 2022 Jul 6;26:202. doi: 10.1186/s13054-022-04061-6 (PMC9258010; doi:10.1186/s13054-022-04061-6)
Supplement: Supplementary file 1 — Additional file 1. Supplementary information on further results. [file 13054_2022_4061_MOESM1_ESM.docx]

Extravascular lung water levels are associated with mortality – A systematic review and meta-analysis

Francesco GAVELLI, MD, PhD^1,2*^; Rui SHI, MD^1,3*^; Jean-Louis TEBOUL, MD, PhD^1,3^; Danila AZZOLINA, PhD^4^; Pablo MERCADO, MD^5^; Mathieu JOZWIAK MD, PhD^6,7^; Michelle S CHEW, MD, PhD^8^; Wolfgang HUBER, MD^9^; Mikhail Y. KIROV, MD, PhD^10^; Vsevolod V. KUZKOV, MD, PhD^10^; Tobias LAHMER, MD^9^; Manu L. N. G. MALBRAIN, MD, PhD^11,12^; Jihad MALLAT, MD^13,14^; Samir G. SAKKA, MD, PhD^15^; Takashi TAGAMI, MD, MPH, PhD^16^; Tài PHAM, MD, PhD^1,17^, Xavier MONNET, MD, PhD^1,3^

1. Service de médecine intensive-réanimation, AP-HP, Hôpital de Bicêtre, DMU CORREVE, Le Kremlin-Bicêtre, France.

2. Emergency medicine Unit, Department of Translational Medicine, Università degli Studi del Piemonte Orientale, Novara, Italy.

3. Université Paris-Saclay, Inserm UMR S_999, FHU SEPSIS, CARMAS, Le Kremlin-Bicêtre, France.

4. Department of Environmental and Preventive Science, University of Ferrara, Ferrara, Italy.

5. Universidad del Desarrollo, Facultad de Medicina Clínica Alemana, Santiago, Chile.

6. Service de Médecine Intensive Réanimation, Centre Hospitalier Universitaire l'Archet 1, 151 route Saint Antoine de Ginestière, 06200 Nice, France.

7. Equipe 2 CARRES, UR2CA - Unité de Recherche Clinique Côte d'Azur, Université Côte d’Azur, Nice, France.

8. Department of Anaesthesia and Intensive Care, Biomedical and Clinical Sciences, Linköping University, Linköping, Sweden.

9. II. Medizinische Klinik und Poliklinik, Klinikum rechts der Isar der Technischen Universität München, Munich, Germany.

10. Department of Anesthesiology and Intensive Care Medicine, Northern State Medical University, Arkhangelsk, Russia.

11. First Department Anaesthesiology and Intensive Therapy, Medical University of Lublin, Jaczewskiego street 8, 20-954 Lublin, Poland.

12. International Fluid Academy, Lovenjoel, Belgium.

13. Department of Anesthesiology and Critical Care Medicine, Schaffner Hospital, Lens, France.

14. Department of Critical Care Medicine, Cleveland Clinic Abu Dhabi, United Arab Emirates.

15. Department of Intensive Care Medicine, Gemeinschaftsklinikum Mittelrhein gGmbH, Academic Teaching Hospital of the Johannes Gutenberg University Mainz, Koblenz, Germany.

16. Department of Emergency and Critical Care Medicine, Nippon Medical School Musashi Kosugi Hospital, Kanagawa, Japan.

17. Université Paris-Saclay, UVSQ Inserm U1018, Equipe d’Epidémiologie respiratoire intégrative, CESP, 94807, Villejuif, France.

* Both authors equally contributed to this work.

**Supplementary material S1**: Searching strategy.

**Supplementary material S2**: PRISMA Checklist.

**Supplementary material S3**: Assessment of risk of bias using the Quality In Prognosis Studies (QUIPS) tool.

**Supplementary material S4**: Mortality rate and extravascular lung water values in included studies.

**Supplementary material S5**: The weighted mortality rates of included studies.

**Supplementary material S6**: Subgroup analysis according to odds ratio on the EVLW reported at baseline *vs.* maximal EVLW.

**Supplementary material S7**: Subgroup analysis according to the mean differences between survivors and non-survivors on the EVLW reported at baseline *vs.* maximal EVLW.

**Supplementary material S8**: Subgroup analysis according to the odds ratio on the EVLW indexed by actual body weight *vs.* predicted body weight.

**Supplementary material S9**: Subgroup analysis according to the mean differences between survivors and non-survivors on the EVLW indexed by actual body weight *vs.* predicted body weight.

**Supplementary material S10**: Subgroup analysis according to odds ratio on study population (ARDS *vs.* non-ARDS population).

**Supplementary material S11:** Subgroup analysis according to the mean differences between survivors and non-survivors on study population (ARDS *vs.* non-ARDS population).

**Supplementary material S12:** Subgroup analysis according to odds ratio on the risk of bias (moderate and low *vs.* high risk of bias).

**Supplementary material S13**: Subgroup analysis according to the mean differences between survivors and non-survivors on the risk of bias (moderate and low *vs.* high risk of bias).

**Supplementary material S14**: Publication bias.

**Supplementary material S1:** Searching strategy

#1 EVLW

#2 EVLWi

#3 #1 OR #2

#4 lung water

#5 #3 OR #4

#5 mortality

#6 survival

#7 #5 OR #6

#8 #5 AND #7

Example, PubMed

((mortality) OR (survival)) AND ((((EVLWI) OR (EVLW)) OR ("lung water"))

Search: ((mortality) OR (survival)) AND ((((EVLWI) OR (EVLW)) OR ("lung water"))

("mortality"[MeSH Terms] OR "mortality"[All Fields] OR "mortalities"[All Fields] OR "mortality"[MeSH Subheading] OR ("mortality"[MeSH Subheading] OR "mortality"[All Fields] OR "survival"[All Fields] OR "survival"[MeSH Terms] OR "survivability"[All Fields] OR "survivable"[All Fields] OR "survivals"[All Fields] OR "survive"[All Fields] OR "survived"[All Fields] OR "survives"[All Fields] OR "surviving"[All Fields])) AND ("EVLWI"[All Fields] OR "EVLW"[All Fields] OR "lung water"[All Fields])

*Translations*

mortality: "mortality"[MeSH Terms] OR "mortality"[All Fields] OR "mortalities"[All Fields] OR "mortality"[Subheading]

survival: "mortality"[Subheading] OR "mortality"[All Fields] OR "survival"[All Fields] OR "survival"[MeSH Terms] OR "survivability"[All Fields] OR "survivable"[All Fields] OR "survivals"[All Fields] OR "survive"[All Fields] OR "survived"[All Fields] OR "survives"[All Fields] OR "surviving"[All Fields]

**Supplementary material S2:** PRISMA Checklist.

| **Section/topic** | **#** | **Checklist item** | **Reported on page #** |
| --- | --- | --- | --- |
| **TITLE** | | |  |
| Title | 1 | Identify the report as a systematic review, meta-analysis, or both. | 1 |
| **ABSTRACT** | | |  |
| Structured summary | 2 | Provide a structured summary including, as applicable: background; objectives; data sources; study eligibility criteria, participants, and interventions; study appraisal and synthesis methods; results; limitations; conclusions and implications of key findings; systematic review registration number. | 2 |
| **INTRODUCTION** | | |  |
| Rationale | 3 | Describe the rationale for the review in the context of what is already known. | 3 |
| Objectives | 4 | Provide an explicit statement of questions being addressed with reference to participants, interventions, comparisons, outcomes, and study design (PICOS). | 3 |
| **METHODS** | | |  |
| Protocol and registration | 5 | Indicate if a review protocol exists, if and where it can be accessed (e.g., Web address), and, if available, provide registration information including registration number. | 4 |
| Eligibility criteria | 6 | Specify study characteristics (e.g., PICOS, length of follow-up) and report characteristics (e.g., years considered, language, publication status) used as criteria for eligibility, giving rationale. | 4-5 |
| Information sources | 7 | Describe all information sources (e.g., databases with dates of coverage, contact with study authors to identify additional studies) in the search and date last searched. | 4-5 |
| Search | 8 | Present full electronic search strategy for at least one database, including any limits used, such that it could be repeated. | 4-5 |
| Study selection | 9 | State the process for selecting studies (i.e., screening, eligibility, included in systematic review, and, if applicable, included in the meta-analysis). | 5 |
| Data collection process | 10 | Describe method of data extraction from reports (e.g., piloted forms, independently, in duplicate) and any processes for obtaining and confirming data from investigators. | 5 |
| Data items | 11 | List and define all variables for which data were sought (e.g., PICOS, funding sources) and any assumptions and simplifications made. | 4-5 |
| Risk of bias in individual studies | 12 | Describe methods used for assessing risk of bias of individual studies (including specification of whether this was done at the study or outcome level), and how this information is to be used in any data synthesis. | 5 |
| Summary measures | 13 | State the principal summary measures (e.g., risk ratio, difference in means). | 6 |
| Synthesis of results | 14 | Describe the methods of handling data and combining results of studies, if done, including measures of consistency (e.g., I^2^) for each meta-analysis. | 6 |
| Risk of bias across studies | 15 | Specify any assessment of risk of bias that may affect the cumulative evidence (e.g., publication bias, selective reporting within studies). | 5 |
| Additional analyses | 16 | Describe methods of additional analyses (e.g., sensitivity or subgroup analyses, meta-regression), if done, indicating which were pre-specified. | 6 |
| **RESULTS** | | |  |
| Study selection | 17 | Give numbers of studies screened, assessed for eligibility, and included in the review, with reasons for exclusions at each stage, ideally with a flow diagram. | 7 |
| Study characteristics | 18 | For each study, present characteristics for which data were extracted (e.g., study size, PICOS, follow-up period) and provide the citations. | 7 |
| Risk of bias within studies | 19 | Present data on risk of bias of each study and, if available, any outcome level assessment (see item 12). | 7 |
| Results of individual studies | 20 | For all outcomes considered (benefits or harms), present, for each study: (a) simple summary data for each intervention group (b) effect estimates and confidence intervals, ideally with a forest plot. | 8-9 |
| Synthesis of results | 21 | Present results of each meta-analysis done, including confidence intervals and measures of consistency. | 7-8 |
| Risk of bias across studies | 22 | Present results of any assessment of risk of bias across studies (see Item 15). | 9 |
| Additional analysis | 23 | Give results of additional analyses, if done (e.g., sensitivity or subgroup analyses, meta-regression [see Item 16]). | 8-9 |
| **DISCUSSION** | | |  |
| Summary of evidence | 24 | Summarize the main findings including the strength of evidence for each main outcome; consider their relevance to key groups (e.g., healthcare providers, users, and policy makers). | 10-11 |
| Limitations | 25 | Discuss limitations at study and outcome level (e.g., risk of bias), and at review-level (e.g., incomplete retrieval of identified research, reporting bias). | 12 |
| Conclusions | 26 | Provide a general interpretation of the results in the context of other evidence, and implications for future research. | 12 |
| **FUNDING** | | |  |
| Funding | 27 | Describe sources of funding for the systematic review and other support (e.g., supply of data); role of funders for the systematic review. | 16 |

*From:* Moher D, Liberati A, Tetzlaff J, Altman DG, The PRISMA Group (2009). Preferred Reporting Items for Systematic Reviews and Meta-Analyses: The PRISMA Statement. PLoS Med 6(6): e1000097. doi:10.1371/journal.pmed1000097

**Supplementary material S3:** Assessment of risk of bias using the Quality In Prognosis Studies (QUIPS) tool.


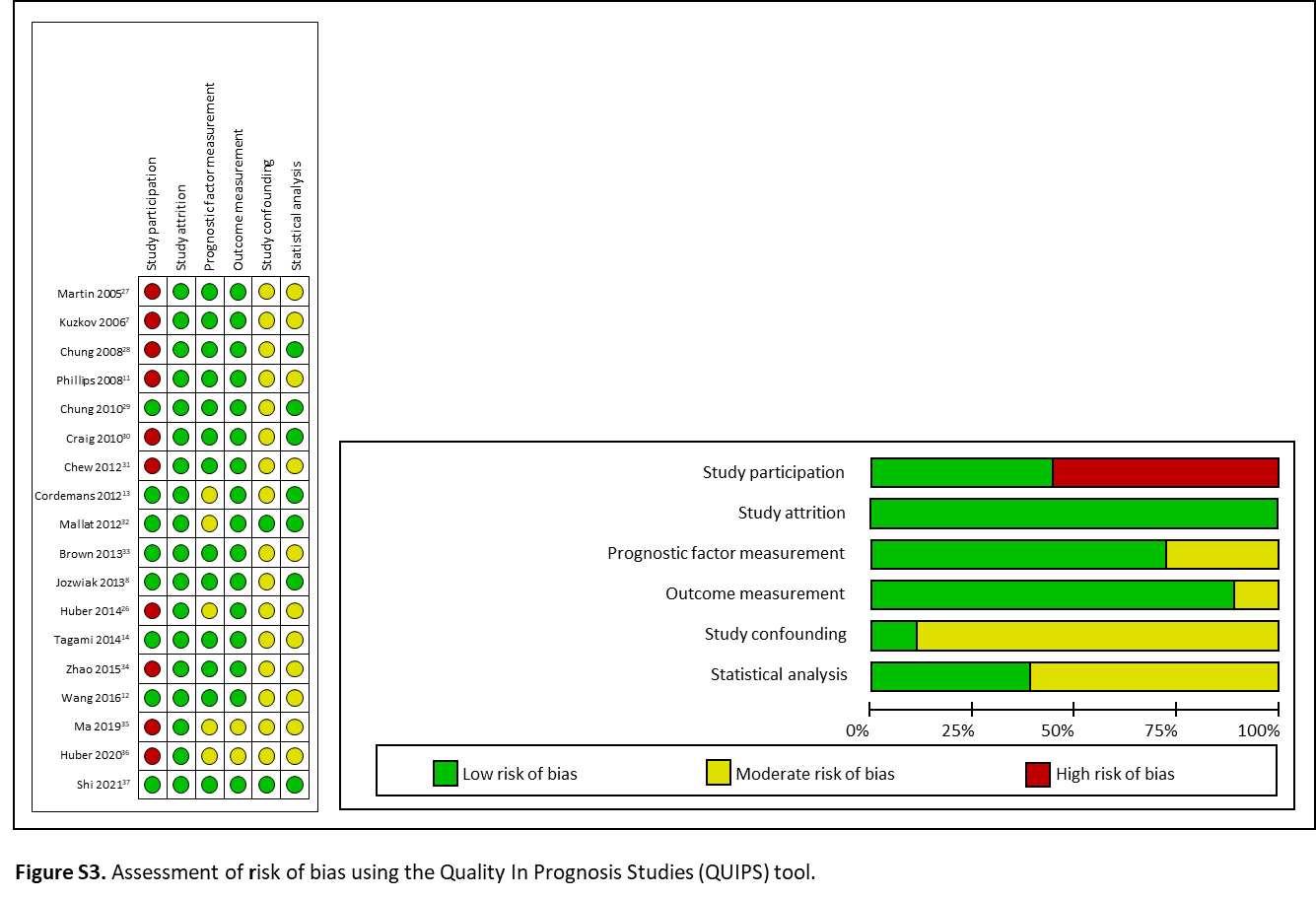


**Supplementary material S4: Mortality rate and extravascular lung water values in included studies.**

| Study ID | N. of patients | Mortality (%) | EVLW baseline non-survivors | EVLW baseline survivors | EVLW max non-survivors | EVLW max survivors |
| --- | --- | --- | --- | --- | --- | --- |
| Martin 2005^27^ | 29 | 41% | 14.0 ± 3.1 | 8.0 ± 3.7 | NA | NA |
| Kuzkov 2006^7^ | 38 | 58% | 9.6 ± 5.0 | 8.3 ± 2.7 | 11.1 ± 6.2 | 8.0 ± 3.0 |
| Chung 2008^28^ | 33 | 52% | 21.0 ± 3.8 | 8.5 ± 1.7 | NA | NA |
| Phillips 2008^11^ | 19 | 37% | 20.6 ± 4.6 | 11.6 ± 1.9 | NA | NA |
| Chung 2010^29^ | 67 | 49% | 24.4 ± 10.6 | 12.2 ± 5.8 | NA | NA |
| Craig 2010^30^ | 44 | 27% | 17.5 ± 2.0 | 10.6 ± 2.4 | NA | NA |
| Chew 2012^31^ | 51 | 29% | 10.6 ± 1.2 | 9.1 ± 1.3 | 13.6 ± 6.7 | 12.4 ± 4.6 |
| Cordemans 2012^13^ | 123 | 53% | 10.5 ± 5.2 | 9.8 ± 3.9 | 13.7 ± 5.9 | 11.7 ± 4.3 |
| Mallat 2012^32^ | 55 | 42% | 15.8 ± 3.3 | 14.5 ± 1.6 | 16.5 ± 3.0 | 14.5 ± 1.6 |
| Brown 2013^33^ | 59 | 30% | 17.0 ± 9.0 | 12.0 ± 5.0 | NA | NA |
| Jozwiak 2013^8^ | 200 | 54% | 17.0 ± 9.0 | 16.0 ± 7.0 | 24.0 ± 10.0 | 19.0 ± 7.0 |
| Huber 2014^26^ | 50 | 32% | NA | NA | NA | NA |
| Tagami 2014^14^ | 192 | 31% | 18.3 ± 6.5 | 18.4 ± 6.7 | 21.8 ± 9.2 | 20.7 ± 7.3 |
| Zhao 2015^34^ | 21 | 38% | 19.0 ± 3.0 | 13.0 ± 3.6 | 19.5 ± 0.5 | 13.0 ± 0.5 |
| Wang 2016^12^ | 105 | 57% | 11.9 ± 4.1 | 11.0 ± 3.6 | 14.4 ± 5.3 | 12.0 ± 4.4 |
| Ma 2019^35^ | 41 | 44% | 13.8 ± 6.1 | 11.2 ± 3.4 | NA | NA |
| Huber 2020^36^ | 49 | 33% | NA | NA | 14.0 ± 9.0 | 10.0 ± 4.0 |
| Shi 2021^37^ | 120 | 61% | 17.0 ± 2.0 | 14.0 ± 2.0 | 23.3 ± 7.7 | 18.7 ± 5.2 |

- EVLW: extravascular lung water (mL/kg); EVLW max: maximum value of extravascular lung water (mL/kg); NA: non-assessed.

**Supplementary material S5:** The weighted mortality rates of included studies.


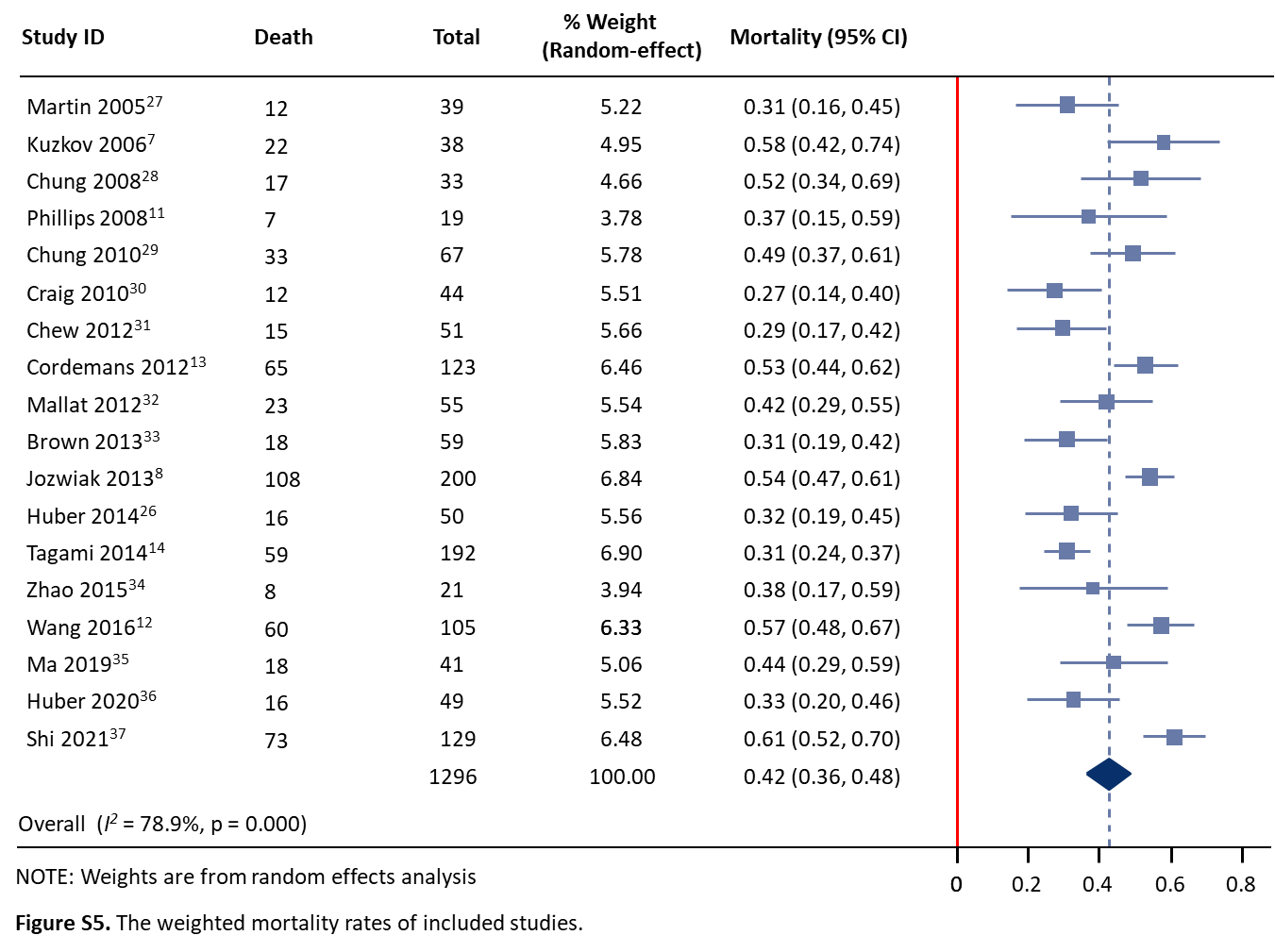


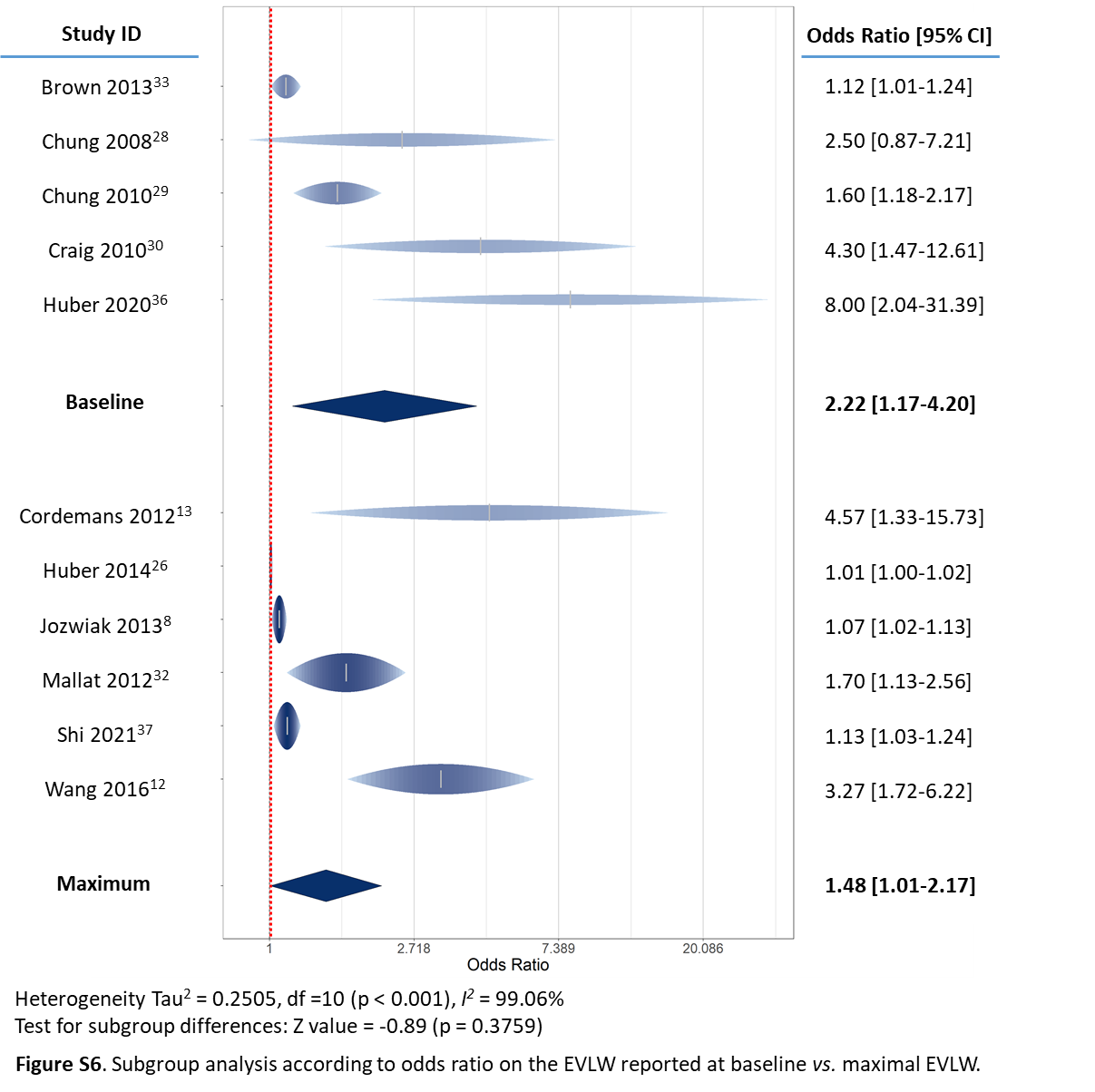
**Supplementary material S6:** Subgroup analysis according to odds ratio on the EVLW reported at baseline *vs.* maximal EVLW.

**Supplementary material S7:** Subgroup analysis according to the mean differences between survivors and non-survivors on the EVLW reported at baseline vs. maximal EVLW.


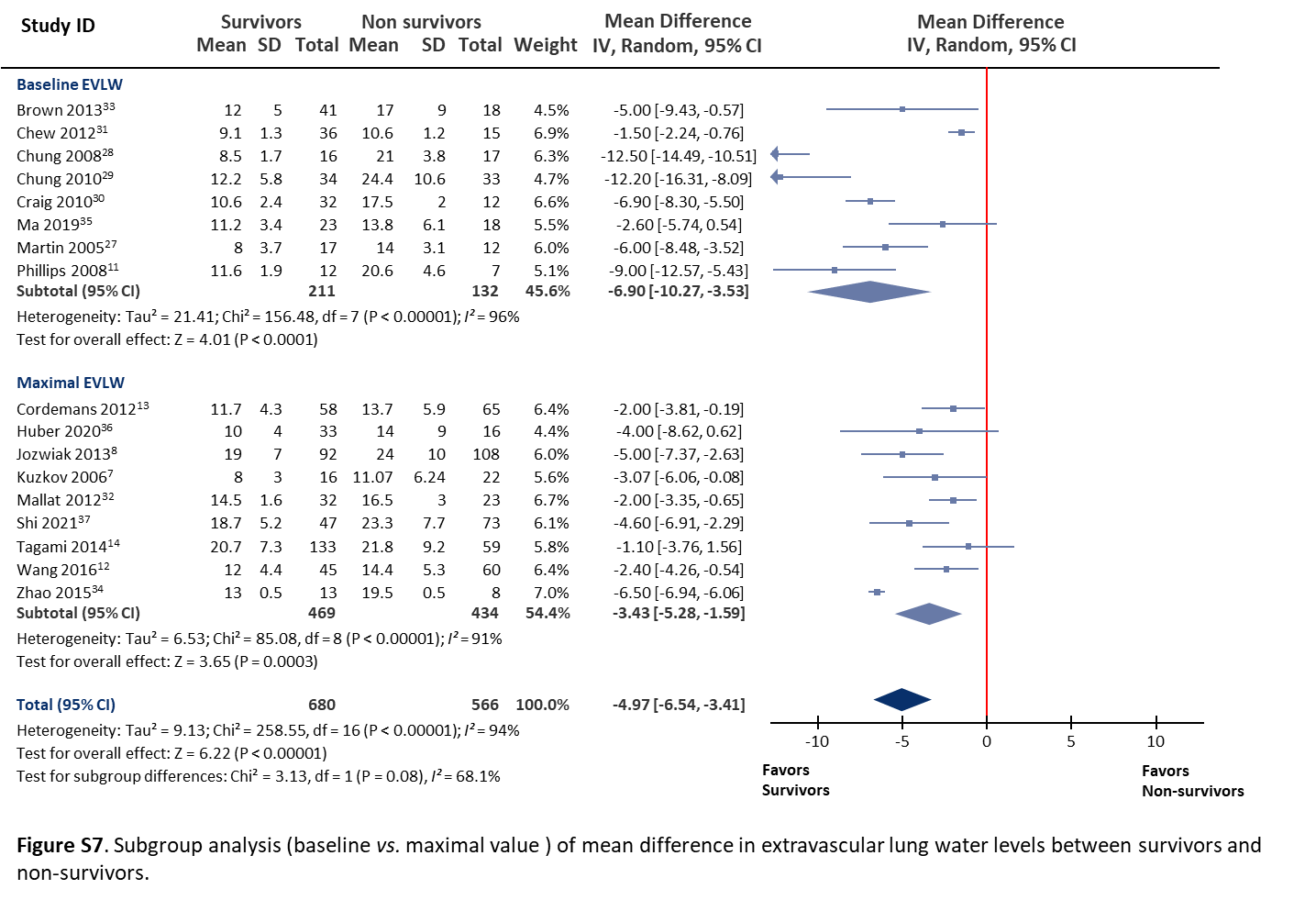


**
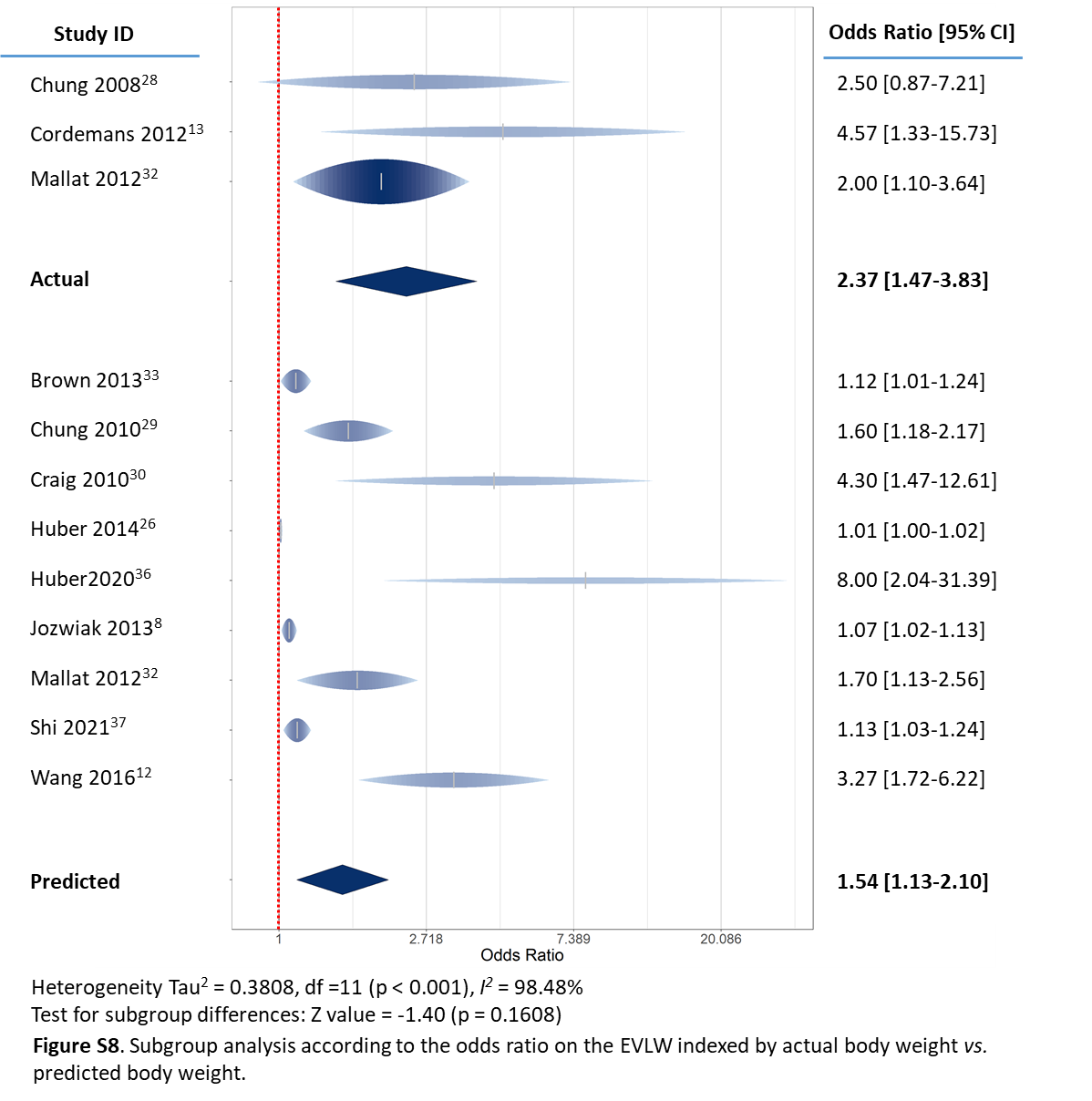
Supplementary material S8:** Subgroup analysis according to the odds ratio on the EVLW indexed by actual body weight *vs.* predicted body weight**.**

**Supplementary material S9:** Subgroup analysis according to the mean differences between survivors and non-survivors on the EVLW indexed by actual body weight vs. predicted body weight


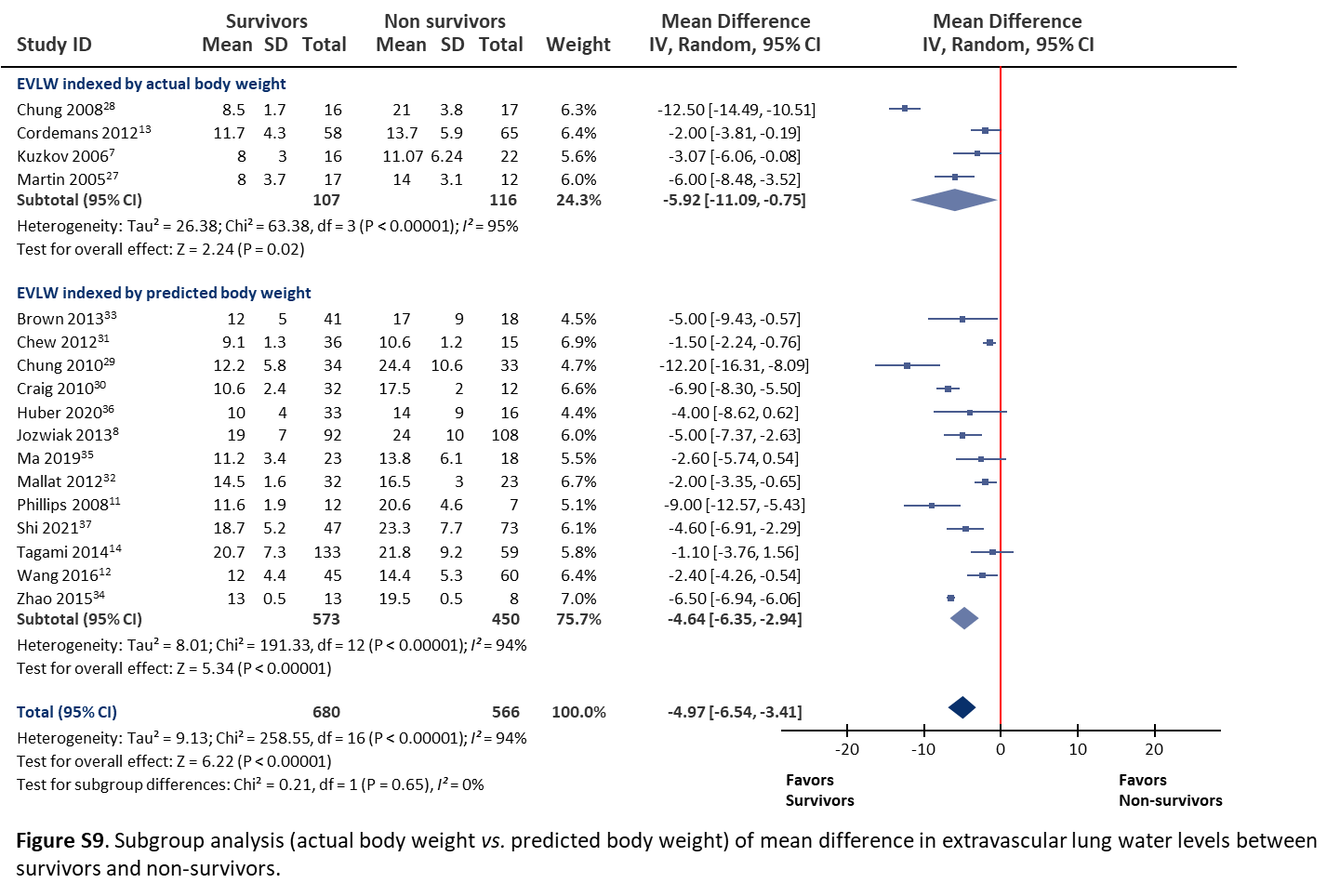
.


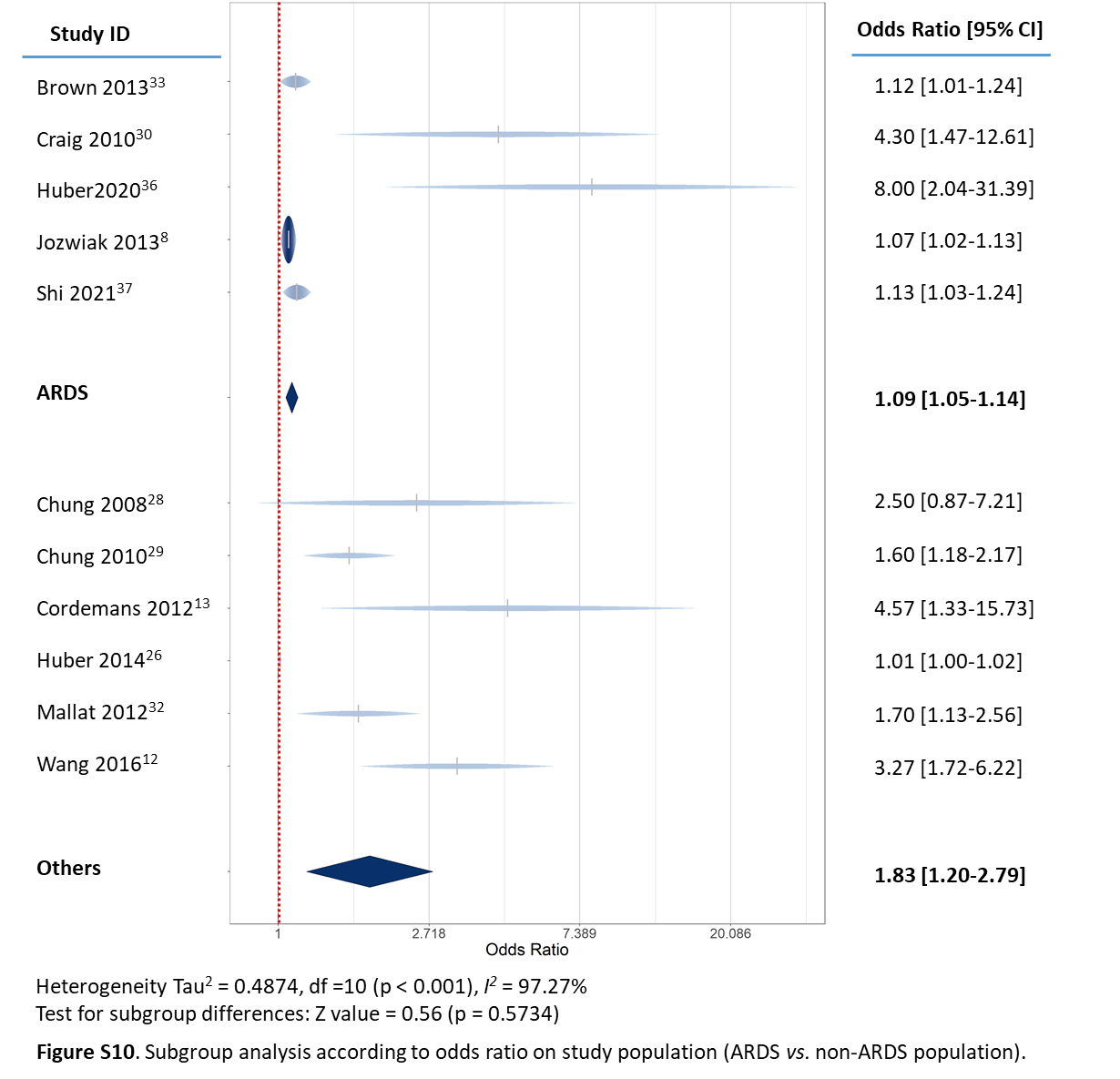
**Supplementary material S10:** Subgroup analysis according to odds ratio on study population (ARDS *vs.* non-ARDS population).

**Supplementary material S11:** Subgroup analysis according to the mean differences between survivors and non-survivors on study population (ARDS *vs.* non-ARDS population).


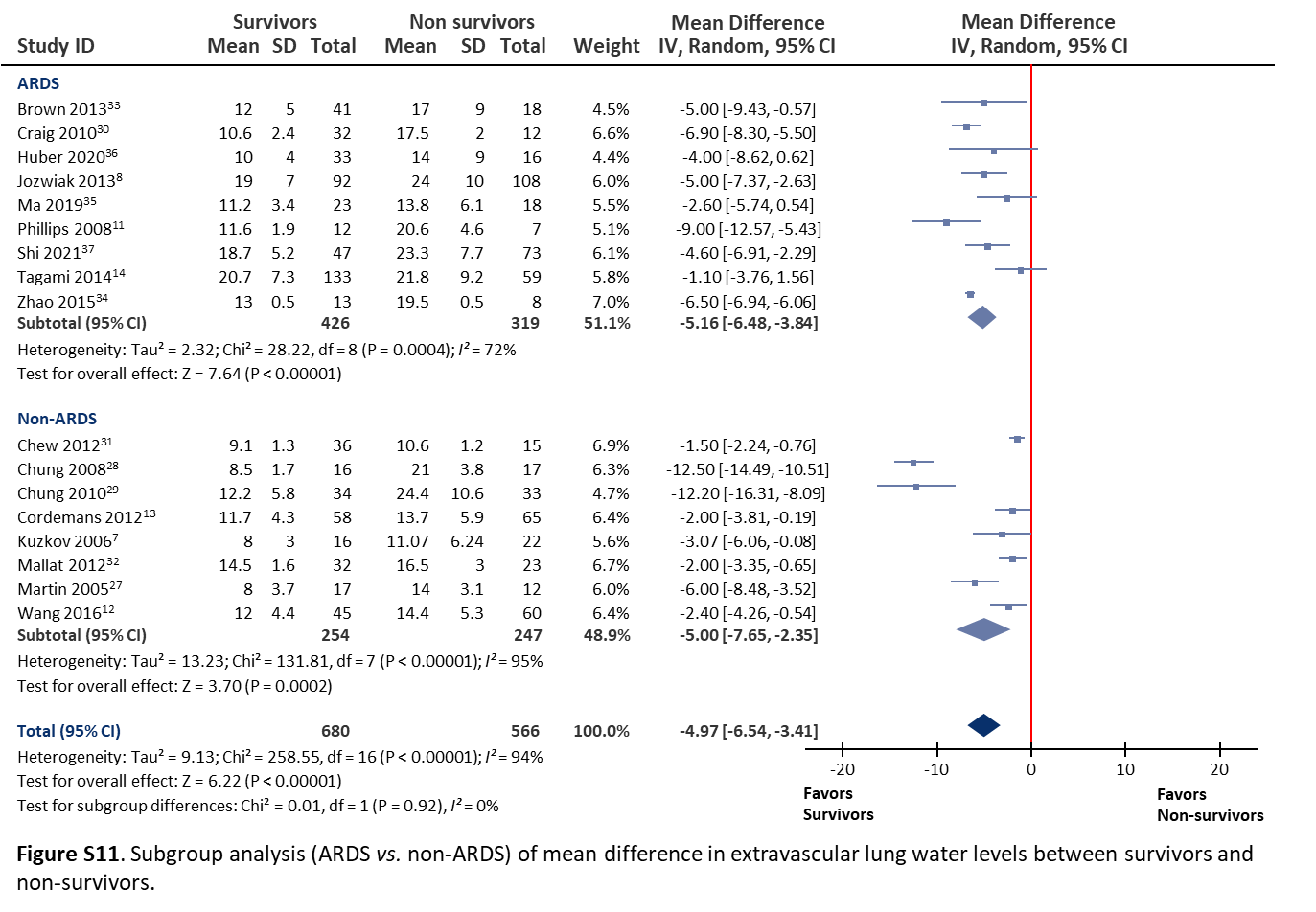


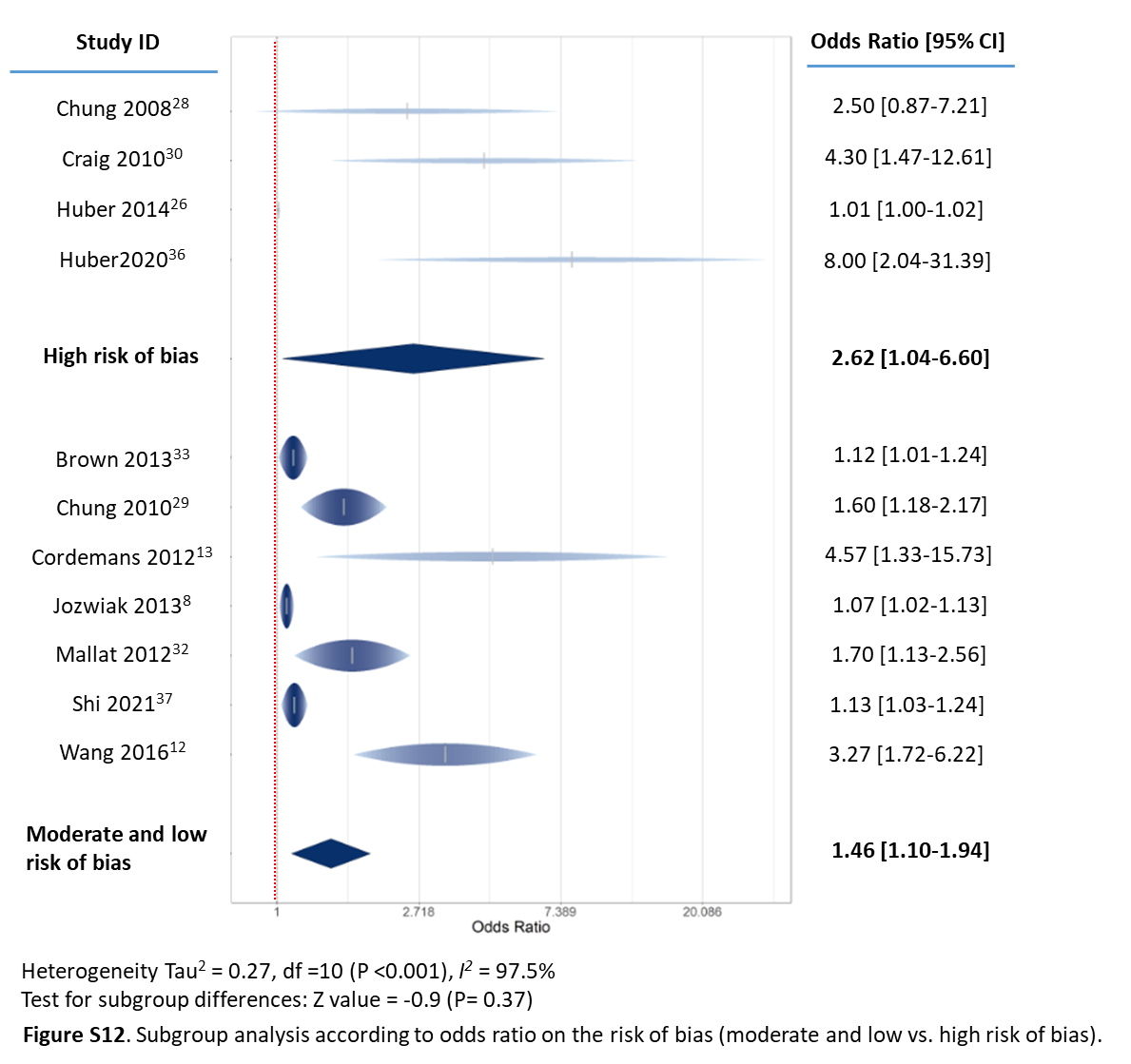
**Supplementary material S12:** Subgroup analysis according to odds ratio on the risk of bias (moderate and low *vs.* high risk of bias).

**Supplementary material S13:** Subgroup analysis according to the mean differences between survivors and non-survivors on the risk of bias (moderate and low *vs.* high risk of bias).


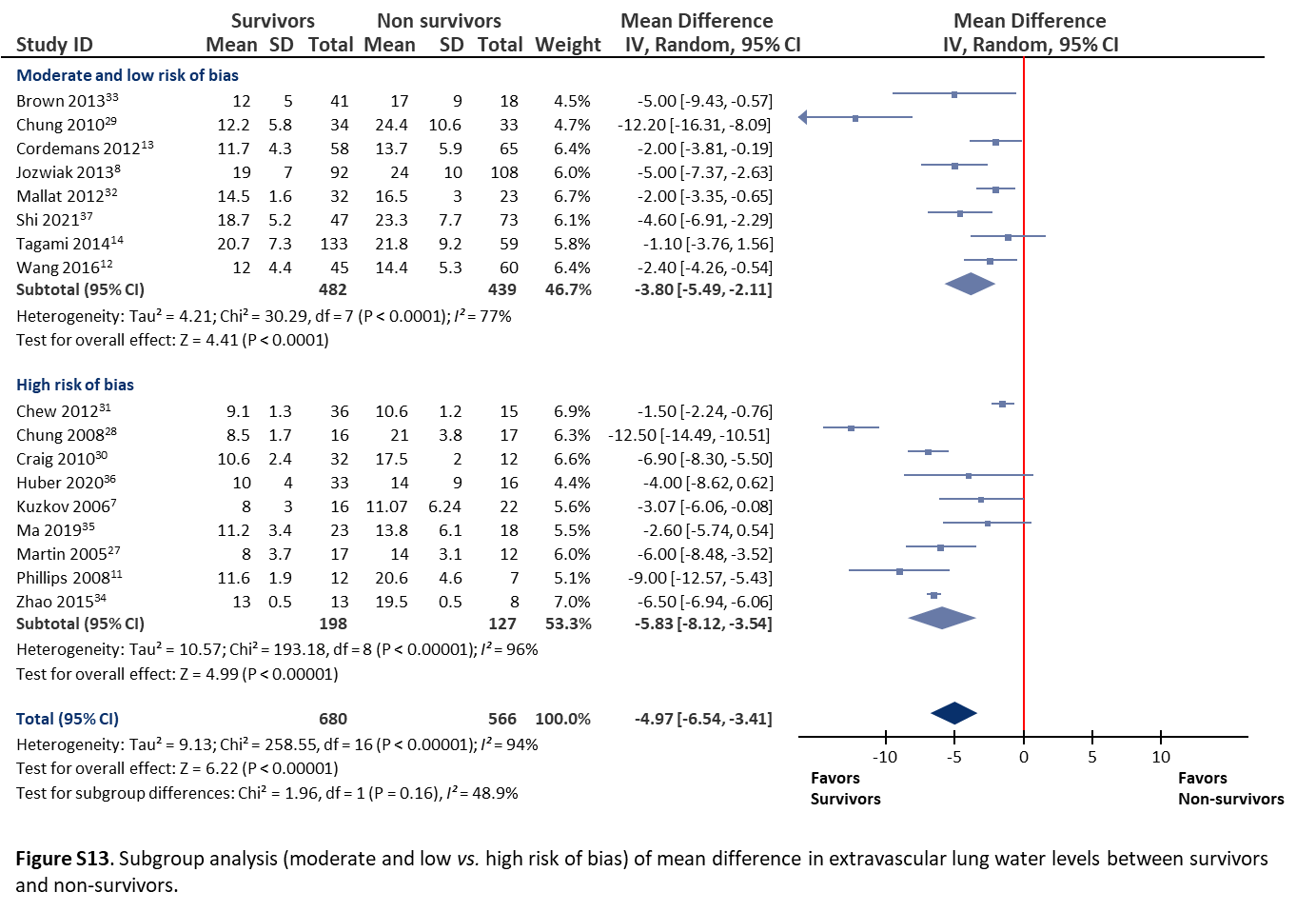


**Supplementary material S14:** Publication bias.

**Linear regression of funnel plot asymmetry**

| **t** | **df** | **Biais** | **SE Biais** | **Intercept** | **p** |
| --- | --- | --- | --- | --- | --- |
| -1.06 | 15 | -1.5456 | 1.4605 | 0.8193 | 0.3056 |


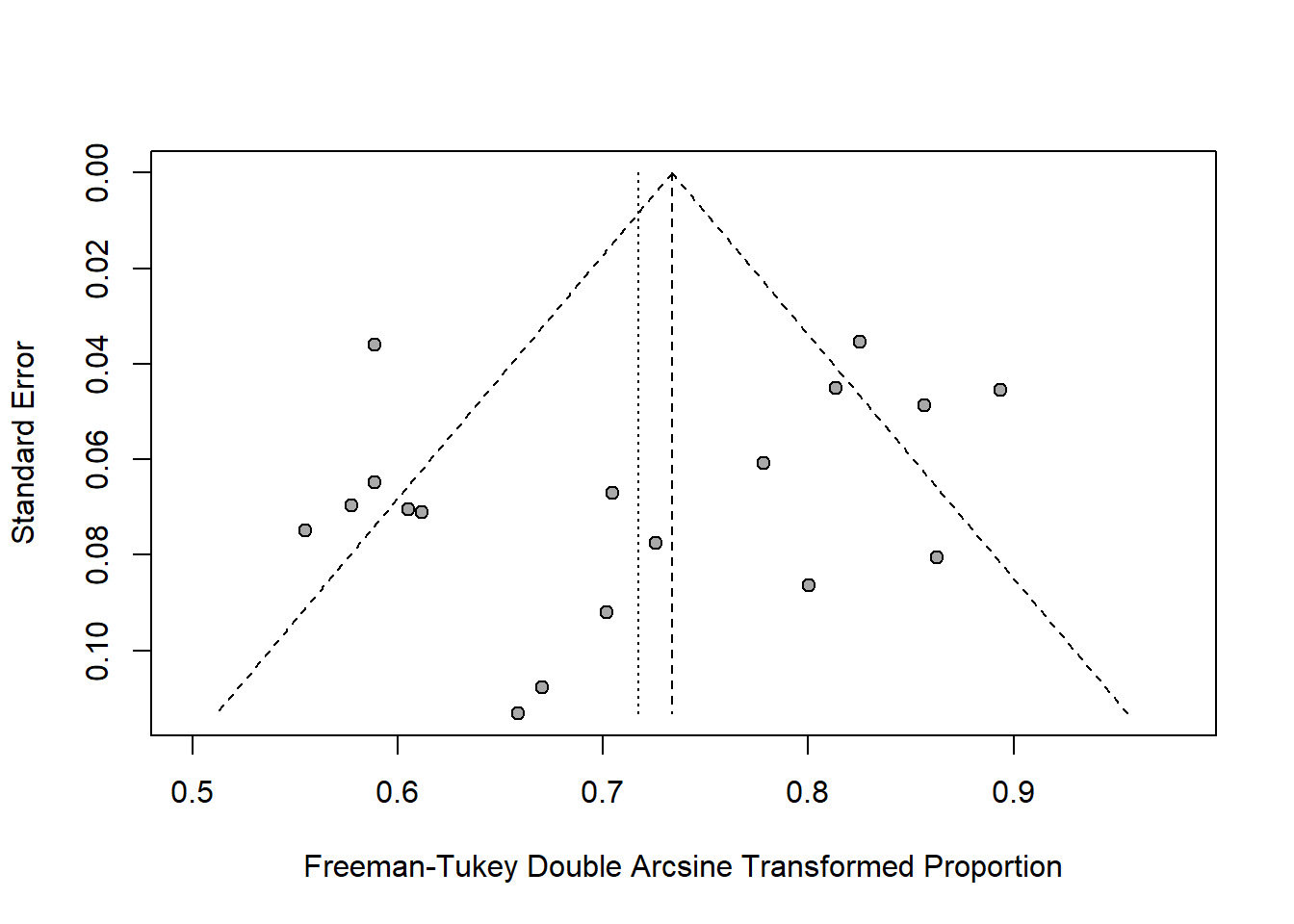
SE: Standard Error
